# Cross-Layer Stream Allocation of mMIMO-OFDM Hybrid Beamforming Video Communications

**DOI:** 10.3390/s25082554

**Published:** 2025-04-17

**Authors:** You-Ting Chen, Shu-Ming Tseng, Yung-Fang Chen, Chao Fang

**Affiliations:** 1 Department of Electronic Engineering, National Taipei University of Technology, Taipei 106, Taiwan; 2 Department of Communication Engineering, National Central University, Taoyuan 320, Taiwan; 3 Faculty of Information Technology, Beijing University of Technology, Beijing 100021, China

**Keywords:** data stream, hybrid beamforming, millimeter-wave (mmWave), multi-user massive multiple-input/multiple-output (MU-Massive MIMO), resource allocation, video distortion, rate-distortion function, broadband access, developing countries

## Abstract

This paper proposes a source encoding rate control and cross-layer data stream allocation scheme for uplink millimeter-wave (mmWave) multi-user massive MIMO (MU-mMIMO) orthogonal frequency division multiplexing (OFDM) hybrid beamforming video communication systems. Unlike most previous studies that focus on the downlink scenario, our proposed scheme optimizes the uplink transmission while also addressing the limitation of prior works that only consider single-data-stream users. A key distinction of our approach is the integration of cross-layer resource allocation, which jointly considers both the physical layer channel state information (CSI) and the application layer video rate-distortion (RD) function. While traditional methods optimize for spectral efficiency (SE), our proposed method directly maximizes the peak signal-to-noise ratio (PSNR) to enhance video quality, aligning with the growing demand for high-quality video communication. We introduce a novel iterative cross-layer dynamic data stream allocation scheme, where the initial allocation is based on conventional physical-layer data stream allocation, followed by iterative refinement. Through multiple iterations, users with lower PSNR can dynamically contend for data streams, leading to a more balanced and optimized resource allocation. Our approach is a general framework that can incorporate any existing physical-layer data stream allocation as an initialization step before iteration. Simulation results demonstrate that the proposed cross-layer scheme outperforms three conventional physical-layer schemes by 0.4 to 1.14 dB in PSNR for 4–6 users, at the cost of a 1.8 to 2.3× increase in computational complexity (requiring 3.6–5.8 iterations).

## 1. Introduction

In response to the increasing demand for higher data transmission rates, the 5G system can utilize the mmWave frequency band to increase available bandwidth [1]. However, the mmWave frequency band poses significant challenges in terms of propagation path loss, susceptibility to blockages, and high penetration loss, making it less reliable for stable communication [2]. To address the propagation path loss issues in the mmWave frequency band, the use of a massive MIMO architecture with multiple antennas has been considered an effective solution.

Beamforming technology is a critical technology in the 5G specification and can be divided into digital beamforming, analog beamforming, and hybrid beamforming (HBF). In terms of bit error rate (BER) performance, digital beamforming offers the best performance. Nevertheless, the hardware cost can be substantially high due to the need to connect each antenna to a RF chain. Moreover, fully digital beamforming requires extensive power consumption, making it impractical for large-scale deployments. Analog beamforming exhibits the poorest performance among the three techniques. It typically relies on hardware phase shifters to adjust the phase and achieve energy concentration in specific directions. However, analog beamforming has lower beam control accuracy and limited adaptability due to its reliance on hardware-based phase adjustments. As a result, neither digital beamforming nor analog beamforming alone is optimal for 5G mmWave applications. Hence, the hybrid beamforming architecture is a good compromise between system performance and hardware cost. It combines high-dimensional phase shifters with low-dimensional digital processors [3]. Hybrid beamforming is particularly advantageous in multi-user (MU) scenarios, where balancing multiple data streams efficiently is crucial. It is notable that the hybrid beamforming structure was already adopted by the current 5G New Radio (NR) systems [4].

In [3,5], the authors studied single-user (SU) and multi-user (MU) mmWave MIMO narrowband single-carrier systems. However, these methods lack the flexibility to adapt to wideband mmWave channels, making them unsuitable for real-world 5G communication systems that require efficient spectrum utilization over broad frequency ranges. In wideband environments, there is a frequency-selective fading issue, which is typically addressed using orthogonal frequency division multiplexing (OFDM) technology [6,7].

In [8], the authors explored the design of HBF for SU-MIMO-OFDM systems. But considering a single user in practical environments is unrealistic, as most 5G applications involve multiple users sharing the network simultaneously. In [9], the authors investigated the HBF design of MU-MIMO-OFDM systems. However, the mobile stations (MS) only employ analog beamforming, which means the base station (BS) can only transmit only one data stream to the MS. This limitation reduces transmission efficiency and prevents the system from leveraging the full potential of multi-user MIMO (MU-MIMO).

When a user has multiple RF chains, the BS has the flexibility to allocate multiple data streams to the user dynamically, taking into account the channel conditions. This dynamic allocation can significantly improve the system performance [10]. The beamforming design in [10] adopted a fully digital beamforming architecture, which means it cannot be directly applied to hybrid beamforming architectures due to hardware constraints and cost limitations. The concept of data stream allocation in hybrid beamforming design has been addressed in related studies such as [11,12,13,14]. However, it is important to note that these studies focus on single-carrier systems. Thus, the applicability of their findings to OFDM-based mmWave systems remains an open question.

In addition to physical layer resource allocation, cross-layer resource allocation recently becoming popular. It is a resource allocation strategy considering the mutual influence between the physical layer channel state information and the application layer video rate distortion (RD) function. Unlike traditional resource allocation approaches that treat the physical and application layers separately, cross-layer optimization provides a holistic approach to maximizing video quality while maintaining efficient spectrum utilization. Different videos have different RD functions, so cross-layer allocation has an inherent advantage in that we allocate more resources to the user whose video has most to gain in video distortion reduction [15]. This strategy is particularly effective in scenarios with dynamic network conditions, where user priorities and network congestion levels fluctuate over time. There have been numerous studies indicating that cross-layer resource allocation can enhance video quality [15,16,17], providing a better simulation of real-world usage scenarios where internet video consumes most internet traffic.

Some interesting real-world usage scenarios include Wireless Multimedia Sensor Networks (WMSNs) [18], where distributed camera and microphone networks generate large volumes of multimedia content, and Internet of Video Things (IoVT) [19], where intelligent transportation systems utilize real-time road monitoring and vehicle-to-vehicle video sharing to enhance driving safety and efficiency. Given the increasing reliance on video-based applications in modern networks, optimizing hybrid beamforming and data stream allocation for cross-layer video communication is an urgent and relevant research challenge.

## 2. Related Works

In [4], the authors considered a downlink MU-MISO system with BS with massive antennas and multiple single-antenna users where a hybrid precoding and simultaneous wireless information and power transfer (SWIPT) are adopted. However, the single-antenna user can support only one data stream and downlink case is considered. In comparison, the proposed scheme considers the multi-antenna user which can support multiple data streams and consider the uplink case.

In [20], data streams are allocated in multi-carrier OFDM systems. Its objective function was sum data rate maximization, in which the downlink case is considered, and only the physical layer metrics are considered. In comparison, the proposed scheme considers the uplink case and the cross-layer performance metric PSNR for video quality.

In [21], multiple data streams are allocated in MU-MIMO-OFDM systems. Its objective function was power consumption minimization, in which the downlink case is considered, and only the physical layer metrics are considered. In comparison, the proposed scheme considers the uplink case and the cross-layer performance metric PSNR for video quality.

Most of the abovementioned studies focused on downlink transmission. The uplink transmission was considered in [22], whereby each MS has the ability to dynamically allocate a varying number of transmission data streams, taking into account the CSI at the physical layer. The objective function is set to maximize the spectral efficiency. The aforementioned prior works, however, considered the physical layer only. In comparison, we propose source encoding rate control and cross-layer data stream allocation considering the cross-layer performance metric PSNR for video quality.

The rationale behind this shift from a physical layer performance metric (such as information rate) to cross-layer performance metric (such as video quality PSNR) and its implications on the network’s overall performance is explained as follows. Cross-layer resource management considers the mutual influence between the physical layer channel state information and the application layer video rate distortion (RD) function. Users’ videos would not have the same RD functions (e.g., fast moving video needs many more bits for the same video distortion), so cross-layer allocation has an inherent advantage in that we can allocate more resources to the user whose video has most to gain in video distortion reduction/PSNR increase [15,16,17]. Considering an average PSNR as the objective function, we can obtain a better video quality and better user experience in video communications, which represents the majority of today’s data traffic [23], including the Internet of Video Things [19]. In summary, for video communications, the increase in the received data rate does not necessarily increase the received video quality (e.g., PSNR) because video quality depends on the source encoding rate control and video content [16,24,25,26,27].

The contributions of this paper are as follows:

1. We propose a novel iterative cross-layer data stream allocation scheme with the objective function being to maximize the average peak signal-to-noise ratio (PSNR). Building upon the physical layer data stream allocation from [14,20,22] as the initialization before iterations, the proposed iterative approach allows the user with the lowest video quality to gain an additional data stream from users with more than one data stream, and improves the average PSNR. The iterative process is repeated until there is no further enhancement. In comparison, the authors of [14,20,22] allocated a data stream with a physical layer, the goal being to maximize the information rate, while there is no iterative process in the proposed cross-layer approach.
2. We jointly adapted the video source encoder rate and cross-layer dynamic data stream allocation with objective of the video content-dependent performance metric PSNR. In comparison, the authors of [14,20,22] did not have source encoding rate control and allocated data streams with a physical layer objective independent of video content, such as information rate. The rationale behind this shift is as follows: For video communications, the increase in the received data rate does not necessarily increase the received video quality (e.g., PSNR), because the video quality depends on the source encoding rate control and video content [16,24,25,26,27].
3. The proposed scheme is a general approach in the sense that it can use any physical layer data stream allocation schemes, including those in [14,20,22]. The simulation results show that the proposed cross-layer scheme outperforms the physical layer scheme in [14,20,22] by 1.14 dB, 0.65 dB, and 1.1 dB for 4 users, respectively, as shown in the PSNR performance
4. We analyze theoretical computational complexity of the proposed cross-layer schemes in big O notation in Section 5. Compared to [22], the computational complexity of the proposed cross-layer scheme is 1.8~2.3 times that of the physical scheme [22] when the number of iterations, L, is 3.6~5.8, as shown in Section 5.

## 3. System Model

### 3.1. mmWave MU Massive MIMO-OFDM System

**Figure 1:**
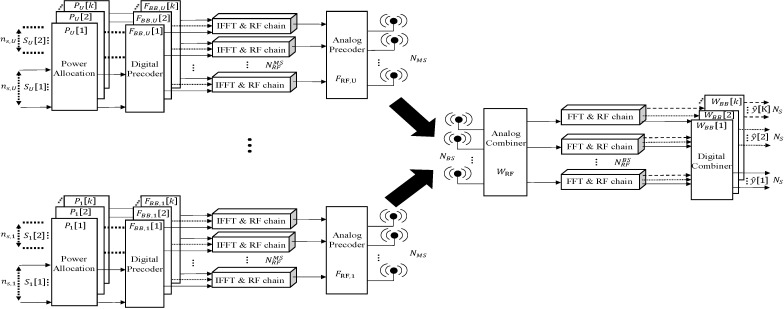
Uplink mm-Wave MU massive MIMO-OFDM system with hybrid beamforming structure.

Figure 1 illustrates the structure of a hybrid beamforming system designed for mmWave MU-Massive MIMO OFDM. The BS is equipped with $N_{BS}$ antennas and $N_{RF}^{BS}$ RF chains, serving U MSs with $N_{MS}$ antennas and $N_{RF}^{MS}$ chains through K subcarriers. In addition, at the BS, the total number of received data streams $N_{S}$ is limited by the number of RF chains of the BS, i.e., $N_{S}$ ≤ $N_{RF}^{BS}$. The system has the capability to allocate varying numbers of transmission data streams to each MS based on their respective CSI.

### 3.2. Channel Model

As in [22], we adopt a cluster channel model with $N_{cl}$ clusters and $N_{ray}$ propagation paths in each cluster, which includes time delays, based on the framework presented in [28] for simulation. According to [29,30], the frequency-domain channel model for the BS and the *u*-th MS on the k-th subcarrier in an OFDM system can be described as follows:

(1) $$H_{u}\left[k\right]=\sqrt{\frac{1}{{PL}_{u}}}\sum_{c=0}^{N_{cl}-1}\sum_{l=1}^{N_{ray}}\alpha_{u,c,l}a_{r}\left(\phi_{u,c,l}^{r}\right)a_{t}^{H}\left(\phi_{u,c,l}^{t}\right)e^{-j2\pi c(k/K)},$$

The path loss is represented by ${PL}_{u}$, while $\alpha_{u,c,l}\sim CN(0,\frac{N_{MS}N_{BS}}{{N_{cl}N}_{ray}})$, $\phi_{u,c,l}^{r}$, and $\phi_{u,c,l}^{t}$ denote the complex path gain, angle of arrival (AOA), and angle of departure (AOD), respectively, for the l-th propagation path in the c-th cluster. The antenna array response vector (AARV) for the receiver and transmitter is denoted as $a_{r}\;$(∙) and $a_{t\;}$(∙), respectively.

### 3.3. Notation

The notation of hybrid beamforming symbols are described in Table 1.

## 4. Proposed Scheme: Cross-Layer Resource Allocation

**Figure 2:**
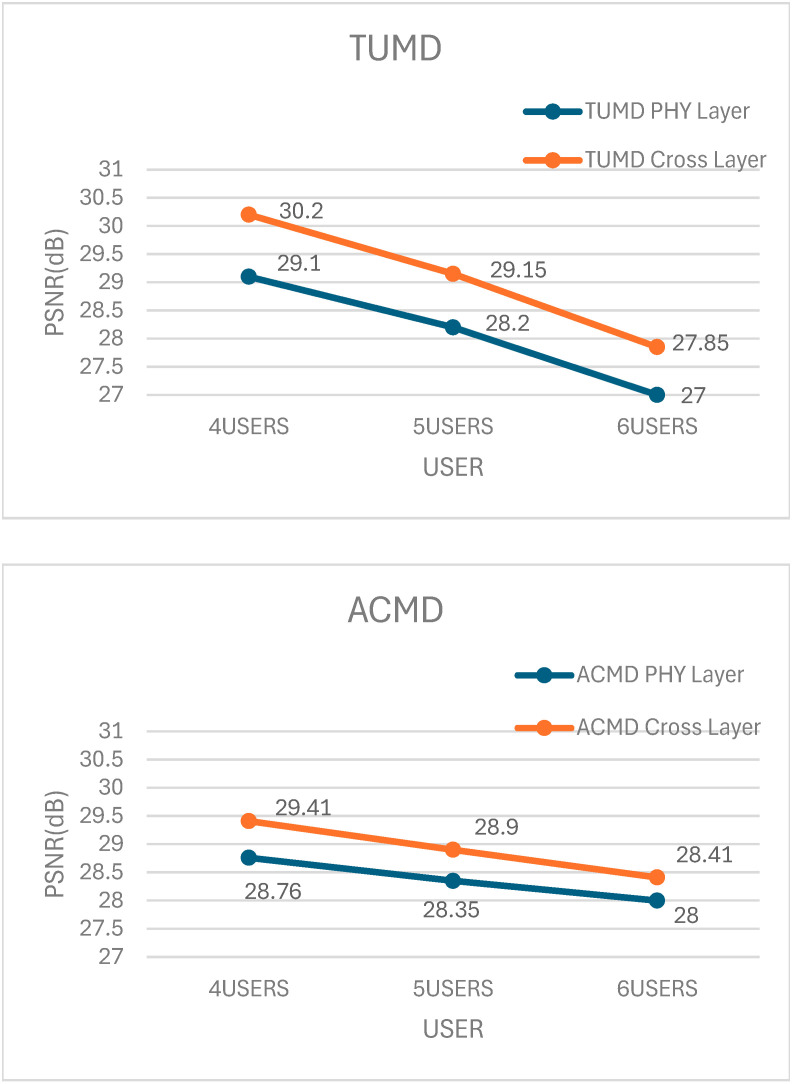

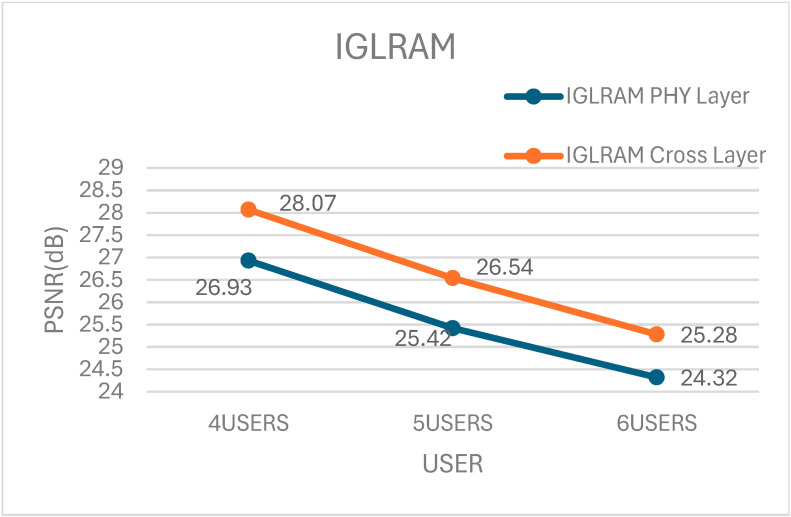
PSNR performance comparisons of the proposed schemes and baseline schemes [14,20,22].

First, we introduce the physical layer baseline scheme in [22], and then our proposed cross-layer scheme, which uses the building blocks of the analog beamformer and digital beamformer in [22] and adds outer iterations allowing lower-PSNR users to contend for data streams with higher-PSNR users to maximize the average PSNR. The proposed cross-layer data stream allocation scheme is a general approach in the sense that it can use any physical layer data stream allocation scheme, not just that of [22], as the starting point before iterations. In Section 6 (Figure 2), we also provide performance comparisons of the cross-layer data stream allocation schemes we are building upon [14,20]. For illustration purposes, we use [22].

### 4.1. Baseline Scheme: Physical Layer Allocation [22]

In [22], a hybrid precoder and combiner is designed for the uplink mmWave MU-Massive-MIMO OFDM system. The objective is to maximize the overall SE while satisfying the constant amplitude constraints and total transmitted power constraint for each MS. To increase the system throughput, multiple users can be served at each subcarrier simultaneously [31], assuming perfect CSI availability. The optimization problem can thus be formulated as follows:

(2) $$\underset{\begin{array}{c} {\left(F_{\mathrm{RF},u}\right)}_{u=1}^{U},{\left[{\left(F_{\mathrm{BB},u}\left[k\right]\right)}_{k=1}^{K}\right]}_{u=1}^{U},\\ W_{\mathrm{RF}},{\left(W_{\mathrm{BB}}\left[k\right]\right)}_{k=1}^{K},\\ {\left(n_{s,u}\right)}_{u=1}^{U},{\left[{\left(P_{u}[k]\right)}_{k=1}^{K}\right]}_{u=1}^{U} \end{array}}{\max}\frac{1}{K}\sum_{k=1}^{K}\sum_{u=1}^{U}\sum_{i=1}^{n_{s,u}}\log_{2}\left(\begin{array}{c} 1+\\ {SINR}_{u,i}\left[k\right] \end{array}\right)$$

subject to

(2a) $$\left|F_{RF,u}(m,n)\right|=\frac{1}{\sqrt{N_{MS}}},\forall u,m,n,$$

(2b) $$\left|W_{RF}\left(m,n\right)\right|=\frac{1}{\sqrt{N_{BS}}},\forall m,n,\;$$

(2c) $$\sum_{k=1}^{K}{\left\|P_{u}\left[k\right]\right\|}_{F}^{2}=P_{0},\forall u,$$

(2d) $${\left\|F_{RF,u}f_{BB,u,i}\left[k\right]\right\|}^{2}=1,\forall u,i,k.$$

Constraints (2a) and (2b) are imposed to ensure constant amplitude on the analog precoder at the MS and the analog combiner at the BS, respectively. Constraint (2c) is imposed to ensure that the total transmit power of the MS is $P_{0}$. To satisfy constraint (2c), the values of $f_{BB,u,i}\left[k\right]$ are normalized in order to meet constraint (2d).

### 4.2. Propose Scheme: Cross-Layer Allocation

Our proposed framework is tailored for video transmission, which takes into account not only the theoretical communication capacity but also the characteristics of the video content and channel propagation conditions. This methodology extends beyond relying solely on physical layer CSI and integrates rate-distortion considerations from the application layer.

The mean square error (MSE) distortion of the *u*-th user can be expressed as

(3) $${MSE}_{u}=a_{u}+\frac{b_{u}}{R_{u}+c_{u}}\;$$

The PSNR of the *u*-th user can be expressed as

(4) $$PSNR_{u}=10\log_{10}\left(\frac{255\times 255}{MSE_{u}}\right)$$

The proposed scheme employs a content-based source encoder rate control similar to that in [15,16,17]. H.264 source encoding with baseline profile is used. The frames inside one Group of Pictures (GOP) are encoded by H.264 source encoding rate control 80, 100, …, 600 kbps. The rate $R_{u}$ (bits/s) in (3) is the target encoding bit rate for the H.264 source encoding rate control.

Our objective is to maximize the average PSNR. $PSNR_{u}$ is the performance metric used to evaluate the video quality of user u, which is inversely proportional to the video MSE distortion ${MSE}_{u}$. In cross-layer resource allocation, maximizing PSNR directly translates to better video quality, making it a more relevant metric than conventional physical-layer objectives such as spectral efficiency. The proposed iterative stream reallocation scheme optimizes PSNR by dynamically adjusting data stream allocation based on the rate-distortion characteristics of each user’s video. Unlike static or purely physical-layer-based allocations, the iterative process allows lower-PSNR users to contend for additional data streams, improving their video quality while maintaining a balance among all users.

The cross-layer resource allocation problem can be expressed as

(5) $$\underset{\begin{array}{c} {\left(F_{\mathrm{RF},u}\right)}_{u=1}^{U},{\left[{\left(F_{\mathrm{BB},u}\left[k\right]\right)}_{k=1}^{K}\right]}_{u=1}^{U},\\ W_{\mathrm{RF}},{\left(W_{\mathrm{BB}}\left[k\right]\right)}_{k=1}^{K},\\ {\left(n_{s,u}\right)}_{u=1}^{U},{\left[{\left(P_{u}\left[k\right]\right)}_{k=1}^{K}\right]}_{u=1}^{U} \end{array}}{\max}\frac{1}{U}\sum_{u=1}^{U}PSNR_{u}$$

where the constraints are the same as those in (2a–d).

The reasons for employing source encoding rate control and the video content-dependent performance metric PSNR is as follows: For video communications, the increase in the received data rate does not necessarily increase the received video quality (e.g., PSNR) because the video quality depends on the source encoding rate control and video content [16,24,25,26,27].

The proposed cross-layer hybrid beamforming scheme uses the building blocks of the analog beamformer Tensor Unfolding Matrix Decomposition (TUMD) and digital beamformer Uplink Coordinated Block Diagonalization (UPCBD) in [22] and adds outer iterations allowing lower-PSNR users to contend for data streams with higher-PSNR users to maximize the average PSNR.

The pseudo-code of the analog beamforming TUMD algorithm proposed in [22] is described in Algorithm 1 below. The algorithm is inspired by tensor-unfolding, which is a mathematical operation that converts a tensor into a matrix, aiming to simplify calculations. The CSI of MIMO-OFDM is represented by a three-dimensional matrix with dimensions ($N_{BS}$*$N_{MS}$*K). By applying tensor unfolding and eigenvalue decomposition (EVD) mathematical operations, the algorithm computes the analog precoder $F_{RF,u}$ and analog combiner $W_{RF}$. Please refer to the paper [22] for a detailed description of the algorithm and its mathematical formulation.
**Algorithm 1:** Analog beamforming design TUMD in [22].**Input:** $H_{u}\left[k\right],\;u=1,\;2,\;\cdots ,\;U,\;k=1,\;2,\;\cdots ,\;K,\;N_{RF}^{MS},\;N_{RF}^{BS}$ 1: **for** u=1 to U **do** 2: $\;H_{u}^{\left(l\right)}={\left[\begin{array}{ccc} {\left(H_{u}[1]\right)}^{T} & {\left(H_{u}[2]\right)}^{T} & \begin{array}{cc} \cdots & {\left(H_{u}[K]\right)}^{T} \end{array} \end{array}\right]}^{T}\in C^{(KN_{BS})\times N_{MS}}$ represents the longitudinal tensor-unfolding of a three-dimensional matrix. 3: $\;\mathrm{Do}\;\mathrm{EVD}.\;{H_{u}^{\left(l\right)}}^{H}H_{u}^{\left(l\right)}={\bar{V}}_{u}{\bar{D}}_{u}{{\bar{V}}_{u}}^{H}$.               Matrix superscript H means Hermitian transpose. ${\bar{D}}_{u}$ is a diagonal matrix. Diagonal entries are the eigenvalues ${\bar{\rho }}_{u}^{\left(1\right)}\cdots {\bar{\rho }}_{u}^{\left(N_{MS}\right)}$. $\;{\bar{V}}_{u}$ refers to the eigenvector associated with ${\bar{\rho }}_{u}^{\left(n\right)}$. 4: $\;F_{RF,u}=\frac{1}{\sqrt{N_{MS}}}e^{j∠{\bar{V}}_{u}\left(:,1:N_{RF}^{MS}\right)}$ 5:    Definition $H_{eq,u}\left[k\right]=H_{u}\left[k\right]F_{RF,u}$ $\;H_{eq,u}^{\left(h\right)}=[H_{eq,u}\left[1\right]\ldots \;H_{eq,u}\left[K\right]\;]\;\in C^{N_{BS}\times (KN_{RF}^{MS})}$ represents the horizontal tensor-unfolding of a three-dimensional matrix. 6: $\;\mathrm{Do}\;\mathrm{EVD}.\;H_{eq,u}^{\left(h\right)}{H_{eq,u}^{\left(h\right)}}^{H}={\bar{U}}_{eq,u}{\bar{D}}_{eq,u}{{\bar{U}}_{eq,u}}^{H}$ ${\bar{D}}_{eq,u}$ is a diagonal matrix. Diagonal entries are the eigenvalues ${\bar{\rho }}_{eq,u}^{\left(1\right)}\cdots {\bar{\rho }}_{eq,u}^{\left(N_{BS}\right)}$. $\;{\bar{U}}_{eq,u}$ refers to the eigenvector associated with ${\bar{\rho }}_{eq,u}^{\left(n\right)}$ 7: **end for** 8: Assign one RF chain to all served MS. $N_{RF,u}=1,\;u=1,\;2,\;\cdots ,\;U$ 9: **repeat** 10: $\;u^{*}=\underset{u\in C}{\mathrm{argmax}}{\;\bar{\rho }}_{eq,u}^{\left(N_{RF,u}+1\right)}.$ $C=\left\{u|N_{RF,u}<N_{RF}^{MS},u\in U\right\},\;U=\left\{1,\;2,\;\cdots ,\;U\right\}.$ 11: $\;N_{RF,u^{*}}=N_{RF,u^{*}}+1$. 12: **until** $\sum_{u=1}^{U}N_{RF,u}=N_{RF}^{BS}$ or C=∅ 13: ${n_{s,u}=N}_{RF,u},\;u=1,\;2,\;\cdots ,\;U.$ 14: **for** u=1 to U **do** 15: $\;W_{RF,u}=\frac{1}{\sqrt{N_{BS}}}e^{j∠{\bar{U}}_{eq,u}\left(:,1:N_{RF,u}\right)}$ 16: **end for** 17: $W_{RF}=\left[\begin{array}{ccc} W_{RF,1} & W_{RF,2} & \begin{array}{cc} \cdots & W_{RF,U} \end{array} \end{array}\right]$ **Output:**
$F_{RF,u},n_{s,u},\;u=1,\;2,\;\cdots ,\;U$ and $W_{RF}$


The pseudo-code of the digital beamforming algorithm UPCBD in [22] is described in Algorithm 2 below. The design concept of this algorithm is to eliminate the inter-user interference and inter-stream interference by utilizing block diagonalization. It is primarily designed based on the channel state information $H_{u}\left[k\right]$, analog precoder $F_{RF,u}$, and analog combiner $W_{RF}$. It mainly involves performing singular value decomposition (SVD). Please refer to paper [22] for a detailed procedure.
**Algorithm 2:** Digital beamforming design UPCBD in [22].**Input:** $n_{s,u},F_{RF,u},\;u=1,\;2,\;\cdots ,\;U$, $W_{RF},$ $\;H_{u}\left[k\right],\;u=1,\;2,\;\cdots ,\;U\;,k=1,\;2,\;\cdots ,\;K$ 1: **for** k=1 to K **do** 2:       **for** u=1 to U **do** 3:           ${\bar{H}}_{u}\left[k\right]=W_{RF}^{H}H_{u\;}[k]F_{RF,u}$ 4:           Do SVD. $\;{\bar{H}}_{u}\left[k\right]={{\bar{U}}_{u}\left[k\right]\bar{\Sigma }}_{u}\left[k\right]{{\bar{V}}_{u}[k]}^{H}$ 5:           Definition ${\hat{F}}_{u}\left[k\right]={\bar{V}}_{u}\left[k\right]\left(:,1:n_{s,u}\right)$ $\;{\overset{̿}{H}}_{u}[k]={\bar{H}}_{u}\left[k\right]{\hat{F}}_{u}\left[k\right]$ 6:       **end for** 7:       **for** u=1 to U **do** 8:             ${\overset{ˇ}{H}}_{u}\left[k\right]=\left[{\overset{̿}{H}}_{1}[k],\cdots ,{\overset{̿}{H}}_{u-1}[k],{\overset{̿}{H}}_{u+1}[k],\cdots ,{\overset{̿}{H}}_{U}[k]\right]$ 9:             Do SVD. $\;{\overset{ˇ}{H}}_{u}\left[k\right]=\left[\begin{array}{cc} {\overset{ˇ}{U}}_{u1}\left[k\right] & {\overset{ˇ}{U}}_{u2}\left[k\right] \end{array}\right]{\overset{ˇ}{\Sigma }}_{u}\left[k\right]{{\overset{ˇ}{V}}_{u}\left[k\right]}^{H}$ 10:             Do SVD. $\;{\overset{ˇ}{U}}_{u2}^{H}\left[k\right]{\overset{̿}{H}}_{u}\left[k\right]={\ddot{U}}_{u}\left[k\right]{\ddot{\Sigma }}_{u}\left[k\right]{{\ddot{V}}_{u}\left[k\right]}^{H}$ 11:             Define the digital precoder in its unnormalized form. $\;{\tilde{F}}_{BB,u}\left[k\right]={\hat{F}}_{u}\left[k\right]{\ddot{V}}_{u1}\left[k\right]=$ $\;\left[{\tilde{f}}_{BB,u,1}\left[k\right],\cdots ,{\tilde{f}}_{BB,u,n_{s,u}}\left[k\right]\right]$ 12:             **for** $i=1\;to\;n_{s,u}$ **do** 13:                   $f_{BB,u,i}\left[k\right]=\frac{{\tilde{f}}_{BB,u,i}\left[k\right]}{\left\|F_{RF,u}{\tilde{f}}_{BB,u,i}\left[k\right]\right\|}$ 14:             **end for** 15:             $F_{BB,u}\left[k\right]=[f_{BB,u,1}\left[k\right],\cdots ,f_{BB,u,n_{s,u}}\left[k\right]]$ 16:             $W_{BB,u}\left[k\right]={\overset{ˇ}{U}}_{u2}\left[k\right]{\ddot{U}}_{u1}\left[k\right]$ 17:       **end for** 18: **end for** **Output: **$F_{BB,u}\left[k\right],\;W_{BB,\;u}\left[k\right],\;u=1,\;2,\;\cdots ,\;U,\;k=1,\;2,\;\cdots ,\;K$

The proposed cross-layer data stream allocation is as follows: Initially, we conduct the baseline physical layer scheme [22], Algorithms 1 and 2. Then, we calculate the PSNR for all users and identify the user with the lowest PSNR. We then create a set C, which includes users with more than one data stream. Next, we proceed with the data stream re-allocation. We attempt to reduce one data stream for the users in set C and increase one data stream for the user with the lowest PSNR. Since the number of data streams for each user has changed, we need to rerun the analog beamforming algorithm and digital beamforming algorithm, and then recalculate the PSNR. The design of the analog combiner in step 15 of Algorithm 1 (analog beamforming) depends on the number of data streams for each user. Therefore, we only need to re-compute the analog beamforming algorithm from steps 14 to 17 in Algorithm 1. If the average PSNR after exchanging the data streams is higher than before, we proceed with the data stream exchange; otherwise, we maintain the initial data stream allocation. Assuming that the average PSNR after exchanging the data streams is higher than before, we will once again identify the user with the lowest PSNR. Repeat the above steps until the termination condition is met, which is when the average PSNR is not higher than before.

The pseudo-code of our proposed cross-layer data stream allocation algorithm is shown in Algorithm 3.
**Algorithm 3:** Proposed cross-layer data stream allocation.Our proposed cross-layer data stream allocation framework is indeed designed with modularity and flexibility in mind, allowing it to be integrated with various physical-layer data stream allocation (beamforming) techniques.Steps 1–4 constitute a replaceable function block for analog/digital beamforming and initial data stream allocation. This function block is agnostic to the specific beamforming algorithm used and can be readily substituted with other advanced hybrid beamforming methods such as IGLRAM [14], ACMD [20], and TUMP [22]. Here, we use the Algorithm 1 analog beamformer TUMD [22] and Algorithm 2 digital beamformer UPCBD [22] as an example. 1: Analog Beamforming Selection: Compute $F_{RF,u},n_{s,u},W_{RF}$ using an analog beamforming algorithm (e.g., Algorithm 1 from TUMP [22]). 2: Digital Beamforming Calculation: Compute $F_{BB,u}\left[k\right],\;W_{BB,u}\left[k\right]\;for\;u=1,\;2,\;\cdots ,\;U$ and k=1, 2, ⋯, K using a digital beamforming algorithm (e.g., Algorithm 2 from TUMP [22]). 3: Power Allocation: Apply water-filling power allocation to optimize power distribution. 4: Calculate SE The following steps represent the newly proposed cross-layer data stream allocation in this paper. 5: Calculate average PSNR 6: Index = min(${PSNR}_{u},\;u=1,\;2,\;\cdots ,\;U$) 7: **repeat** 8:       **for** u=1 to U **do** 9:             **if**      $N_{s,u}$ > 1 10:                         add u to Set C 11:             **end if** 12:       **end for** 13:       **for** u=1 to U **do** 14:               **if** $C_{1,u},\;u=1,\;2,\;\cdots ,\;U$ ~= 0 15:                         $N_{s,Index}$ ← $N_{s,Index}$ + 1 16:                         $N_{s,u}$ ← $N_{s,u}$ − 1 17:                         Re-execute Analog/Digital Beamforming (e.g., Algorithms 1 and 2 from TUMP [22])) 18:               **end if** 19:               **if** new average PSNR > previous average PSNR 20:                         data stream change 21:                         Index = min(${PSNR}_{u},u=1,2,\cdots ,U$) 22:               **else** 23:                         $N_{s,Index}$ ← $N_{s,Index}$ − 1 24:                         $N_{s,u}$ ← $N_{s,u}$ + 1 25:               **end if** 26:       **end for** 27: **until** new average PSNR <= previous average PSNR

## 5. Computational Complexity

According to the complexity analysis in [22], the computational complexity of analog beamforming mainly arises from lines 3, 5, and 6 of Algorithm 1. These three lines are executed U times according to the number of users U, resulting in a total complexity of adjusted PSNR. Scheme C has a latency of 2, and Scheme B has latency of 6, so Scheme C outperforms Scheme B in terms of latency.

$$O\left(U\left(2K{N_{BS}N_{MS}N}_{RF}^{MS}+min\left\{{N_{BS}}^{2}KN_{RF}^{MS},N_{BS}K^{2}{\left(N_{RF}^{MS}\right)}^{2}\right\}\right)\right)$$

According to the complexity analysis in [22], the computational complexity of digital beamforming primarily arises from lines 3, 5, 10, 11, and 16 of Algorithm 2. These five lines are executed U times according to the number of users U and K times according to the number of subcarriers K, resulting in a total complexity of

$$O\left(UKN_{BS}N_{RF}^{MS}\left(N_{RF}^{BS}+N_{MS}\right)\right)$$

In our proposed data stream allocation (Algorithm 3), when the data streams are re-allocated, it is necessary to re-compute the analog beamforming algorithm from line 14 to line 17 in Algorithm 1, as well as the complete digital beamforming (Algorithm 2). The analog beamforming (Algorithm 1) only needs to execute from line 14 to line 17 because the design of the analog combiner is dependent on the allocation of user RF chains. Hence, it is sufficient to execute line 14 to line 17 concerning the design of the analog combiner. We assume a variable L to count the number of times the digital beamforming algorithm is re-executed from scratch in the cross-layer data stream allocation algorithm. Since the computational complexity of analog beamforming mainly arises from lines 3, 5, and 6 of Algorithm 1, the computational complexity of analog beamforming can be considered as negligible or close to 0. The computational complexity of digital beamforming increases with the number of iterations of the cross-layer data stream allocation algorithm. The computational complexity of digital beamforming becomes

$$O\left(\;(UKN_{BS}N_{RF}^{MS}\left(N_{RF}^{BS}+N_{MS}\right))\times (1+L)\right).$$

According to the above explanation, the complexity of the cross-layer data stream allocation algorithm we proposed can be summarized as $O\left(U\left(2K{N_{BS}N_{MS}N}_{RF}^{MS}+min\left\{{N_{BS}}^{2}KN_{RF}^{MS},N_{BS}K^{2}{\left(N_{RF}^{MS}\right)}^{2}\right\}\right)\right)$ + $O\left(\;(UKN_{BS}N_{RF}^{MS}\left(N_{RF}^{BS}+N_{MS}\right))\times (1+L)\right)$. Table 2 presents the complexity analysis expressions for both the PHY layer and cross-layer scenarios.

## 6. Simulation Results

This section presents our experimental results. Our simulations were conducted on a computer environment with an 8-core CPU: Intel^®^ Core™ i7-9700 (Intel, Santa Clara, CA, USA) and 48GB of memory.

For video communications, we use a similar setup to that in [15,16,17]. We utilize a sequence of CIF videos with a total duration of 50 s, each containing 30 frames per second. We applied compression using the baseline profile of the H.264/AVC reference software, specifically Version JM 11.0. Our Group of Pictures (GOP) configuration consisted of 15 frames, structured as I-P-P-P, and the frames within each GOP were encoded using H.264 source encoding rate control.

To assess various bitrates, we encoded each GOP at discrete rates between 80 and 600 kbps. These operational points were then used to fit the RD function in (3) through a nonlinear regression approach [15,16,17].

For our resource management, we randomly assigned different starting points of the same video to different users [15,16,17]. The resource allocation decision was made for each GOP, ensuring that the cross-layer resource allocation problem in (5) is optimized.

The hybrid beamforming architecture and parameters of our MU-MIMO OFDM system are based on the environment configuration in [22], as shown in Table 3, where the system being analyzed operates with a total channel bandwidth of 50 MHz and utilizes an OFDM system with 16 subcarriers. Additionally, for all schemes, the optimal water-filling power allocation method is employed for each user (MS).

We conducted experiments with 4, 5, and 6 users, respectively. The BS is equipped with eight RF chains, indicating that the BS can receive a maximum of eight data streams. Each MS has three RF chains, meaning that each MS can receive a maximum of three data streams.

The comparisons of simulation results for PSNR performance for the proposed schemes and baseline schemes [14,20,22] are shown in Figure 2. We have utilized three baseline physical schemes with the same digital beamformer (UPCBD) as that in [22], and three analog beamforming algorithms, namely TUMD [22], Average Channel Matrix Decomposition (ACMD) [20], and Iterative Generalized Low Rank Approximation of Matrices (IGLRAM) [14]. For each baseline PHY layer scheme, we have utilized corresponding proposed cross-layer schemes by substituting the analog beamformer TUMD (Algorithm 1) with ACMD [20] or IGLRAM [14] in Algorithm 3.

The results in Figure 2 represent the outcomes of 10,000 experimental trials. For scenarios with user counts of 4, 5, and 6, the PSNR improvements were as follows: in the TUMD algorithm, an increase of 1.1 to 0.85 dB; in the ACMD algorithm, an enhancement of 0.65 to 0.41 dB; and in the IGLRAM algorithm, a gain of 1.14 to 0.96 dB. It demonstrates the applicability of our proposed cross-layer data stream allocation algorithm to other analog beamforming algorithms as well, not just TUMD in [22].

From the results in Figure 2, it can be observed that as the number of users increases, the improvement in average PSNR becomes less significant. It can be explained as follows: In a scenario with four users and the condition that each user has at least one data stream, there is a higher probability of a situation where a user with one data stream competes with users having three data streams to acquire additional data streams. In a scenario with six users, the probability of a user with one data stream competing against users with three data streams to acquire additional data streams is relatively low. Therefore, the improvement in PSNR for four users is higher compared to that for five users and five users.

The parameters from Table 3 are substituted into the complexity Big O notation in Table 2, and the results are presented in Table 4. In the complexity Big O notation of our proposed cross-layer data stream allocation, the time complexity of analog beamforming is the same as the baseline approach [22], while the time complexity of digital beamforming is 1 + L times that of the baseline approach [22]. L represents the average number of times the digital beamforming is re-executed in the simulation of the cross-layer data stream allocation algorithm. The hybrid beamforming architecture and parameters of the MU-MIMO-OFDM system were set according to the configuration described in Table 3 of the referenced paper [22]. According to our simulation results, L is approximately 5.8 times for four users, 5.2 times for five users, and 3.6 times for six users. Based on the simulation results, it can be observed that L decreases as the number of users increases. The reason is that the number of L is determined by the set C created in line 10 of Algorithm 3. The set C represents the collection of users with more than one data stream. We require each user to have a minimum of one data stream and a maximum of three data streams. Therefore, in the case of four users, set C can only have three or two users; and in the case of six users, set C can only have two or one users. Hence, as the number of users increases, L decreases.

Table 5 presents the results of the simulated elapsed times for the proposed cross-layer data stream allocation algorithm compared to the baseline [22] physical-layer data stream allocation algorithm. From the results in Table 4 and Table 5, it can be observed that the outcomes are roughly consistent, with cross-layer elapsed time being approximately twice that of the PHY layer elapsed. If the number of users increases to six, the elapsed time ratio decrease to approximately 1.6.

## 7. Conclusions

We propose a novel and modular cross-layer data stream allocation framework for uplink mmWave MU-Massive MIMO OFDM hybrid beamforming video communication systems. Unlike conventional approaches that focus solely on physical-layer CSI, our method integrates cross-layer optimization [32,33] by dynamically reallocating data streams from users with more than one stream to the user with the lowest PSNR. This iterative process, driven by both CSI and application-layer PSNR, aims to maximize the average video quality across all users. To ensure flexibility, the first four steps of our revised Algorithm 3 are formulated as a functional block responsible for analog/digital beamforming and initial stream allocation. This block is independent of any specific physical-layer method and can be replaced with various hybrid beamforming algorithms such as IGLRAM [14], ACMD [20], and TUMP [22]. Our simulation results demonstrate that the proposed framework yields significant PSNR improvements across diverse user scenarios (as shown in Table 4) while maintaining a reasonable computational cost of approximately 1.8–2.3× that of conventional methods (Table 5). Furthermore, the modular nature of our design allows for future integration with other physical-layer schemes, including alternative hybrid beamforming and detection techniques, which could serve as additional baselines for comparison.

## 8. Future Directions

A promising future direction is the integration of deep learning-based cross-layer dynamic data stream allocation to further reduce computational complexity. Since the proposed scheme is iterative, the number of iterations can become large when the number of RF chains and users increases. A deep learning-based approach could learn optimal allocation strategies from data, reducing the need for extensive real-time computations while maintaining a high PSNR performance. However, to effectively integrate deep learning into our existing cross-layer framework, further investigations are required to address challenges such as model training, real-time adaptability, and deployment in practical systems. Ensuring that deep learning models generalize well across different network conditions and maintaining low-latency inference are key aspects to be explored in future research.

## Figures and Tables

**Table 1:** Hybrid beamforming symbol description.

Symbol	Quantity
U	number of users.
K	number of subcarriers in OFDM.
$N_{BS}$	number of antennas in a BS.
$N_{RF}^{BS}$	number of RF chains in a BS.
$N_{s}$	number of data streams that the BS can receive.
$N_{MS}$	number of antennas configured on the MS.
$N_{RF}^{MS}$	number of RF chains configured on MS.
$n_{s,u}$	*s*-th data stream transmitted by the *u*-th MS.
$s_{u}\left[k\right]$	transmitted data symbol vector of the *u*-th MS at the k-th subcarrier.
$P_{u}\left[k\right]$	transmit power of the *u*-th MS at the k-th subcarrier.
$F_{BB,u}\left[k\right]$	digital precoder of the *u*-th MS at the k-th subcarrier.
$F_{RF,u}$	analog precoder at user *u*.
$W_{RF}$	analog combiner.
$W_{BB,u}\left[k\right]$	the digital combiner of the *u*-th MS at the k-th subcarrier.
$\hat{y}\left[k\right]$	final processed signal at the k-th subcarrier.

**Table 2:** Complexity comparison.

PHY Layer	$O\left(U\left(2K{N_{BS}N_{MS}N}_{RF}^{MS}+min\left\{{N_{BS}}^{2}KN_{RF}^{MS},N_{BS}K^{2}{\left(N_{RF}^{MS}\right)}^{2}\right\}\right)\right)$ **+** $O\left(UKN_{BS}N_{RF}^{MS}\left(N_{RF}^{BS}+N_{MS}\right)\right)$
Cross-Layer	$O\left(U\left(2K{N_{BS}N_{MS}N}_{RF}^{MS}+min\left\{{N_{BS}}^{2}KN_{RF}^{MS},N_{BS}K^{2}{\left(N_{RF}^{MS}\right)}^{2}\right\}\right)\right)$+ $O\left(\;(UKN_{BS}N_{RF}^{MS}\left(N_{RF}^{BS}+N_{MS}\right))\times (1+L)\right)$

**Table 3:** Hybrid beamforming setup parameters.

Symbol	Quantity
*U*	4, 5, 6
*K*	16
$N_{BS}$	128
$N_{RF}^{BS}$	8
$N_{s}$	8
$N_{MS}$	16
$N_{RF,u}$	1, 2, 3
$n_{s,u}$	1, 2, 3
*BW*	50 MHz

**Table 4:** The actual computational complexity of the proposed schemes and baseline schemes.

User	L	Cross-Layer Data Stream Allocation Computational Complexity Big O (A)	[22] Physical Layer Computational Complexity Big O (B)	Times (A)/(B)
4	5.8	5,976,883.2	2,555,904	2.3
5	5.2	7,028,736	3,194,880	2.2
6	3.6	7,018,905.6	3,833,856	1.8

**Table 5:** Elapsed times.

User	Cross-Layer Data Stream Allocation Elapsed Time(s) (C)	[22] Physical Layer Elapsed Time(s) (D)	Elapse Times Ratio (C)/(D)
4	1439.7 s	689.9 s	2.1
5	1690.4 s	847.4 s	2
6	1644.8 s	1006.9 s	1.6

## Data Availability

The data are available upon request.
